# Synthesis, Physical and Ion-Conducting Properties of 1,2,3-Triazolium Ionic Liquids

**DOI:** 10.3390/molecules31060936

**Published:** 2026-03-11

**Authors:** Imen Abdelhedi Miladi, Maha Chikhaoui, Malak Alaa Eddine, Anatoli Serghei, Hatem Ben Romdhane, Eric Drockenmuller

**Affiliations:** 1 Université de Tunis El Manar, Faculté des Sciences de Tunis, Laboratoire de Chimie (Bio)Organique Structurale et de Polymères (LR99ES14), El Manar, Tunis 2092, Tunisiahatem.benromdhane@fst.utm.tn (H.B.R.); 2 Université Claude Bernard Lyon 1, CNRS, Ingénierie des Matériaux Polymères, UMR 5223, 69622 Villeurbanne, France; malak.alaa-eddine@univ-lyon1.fr (M.A.E.);

**Keywords:** ionic liquid, click chemistry, azide–alkyne 1,3-dipolar cycloaddition, *N*-alkylation, 1,2,3-triazole, 1,2,3-triazolium, ion-exchange, ionic conductivity

## Abstract

1,4-Disubstituted 1,2,3-triazoles are readily obtained by copper(I)-catalyzed azide–alkyne 1,3-dipolar cycloaddition (CuAAC)—the most widespread illustration of click chemistry to date. 1,2,3-Triazoles form a vast and easily accessible library of precursors for synthesizing 1,2,3-triazolium ionic liquids (TILs). A series of four 1,3,4-trisubstituted TILs with *N*-1-*n*-octyl, *N*-3-methyl and different *C*-4 substituents (i.e., aromatic, aliphatic, PEGylated and perfluorinated groups) is synthesized in two steps involving: (i) CuAAC to generate 1,2,3-triazole precursors and (ii) *N*-alkylation of the 1,2,3-triazole groups with methyl iodide to afford the corresponding 1,2,3-triazolium salts with iodide counter-anions. A thorough investigation of the correlations between structure and properties is carried out using NMR spectroscopy, high-resolution mass spectrometry, differential scanning calorimetry, thermogravimetric analysis and broadband dielectric spectroscopy. The PEGylated TIL has also undergone ion metathesis to produce the TIL analogue with a bis(trifluoromethylsulfonyl)imide counter-anion. Of all the synthesized TILs, this derivative exhibits the lowest glass transition temperature (*T*_g_ = −76 °C), the highest thermal stability (*T*_d10_ = 345 °C) and the greatest ionic conductivity (σ_DC_ = 6.5 × 10^−4^ S cm^−1^ at 30 °C under anhydrous conditions).

## 1. Introduction

Ionic liquids (ILs) are organic salts that melt at temperatures below 100 °C [1,2]. Their many appealing features include negligible vapor pressure, wide usable temperature range, broad structural design, low flammability, large electrochemical stability window, and enhanced ionic conductivity, which has generated strong interest in academia and industry [3,4]. ILs have been used as solvents for catalysis [5], as task-specific organocatalysts [6], as well as key components in separation technologies [7], gas separation membranes [8], energy storage [9,10], propellant fuels [11] and pharmaceutics [12]. In addition, the spectacular development of a myriad of poly(ionic liquids) (PILs) is testament to the attention that ILs have attracted from polymer materials scientists [13,14,15]. The versatile functionality, broad structural design and idiosyncratic properties of ILs stem from the vast array of possible combinations of cations (e.g., ammonium, boronium, sulfonium, phosphonium, guanidinium, pyrrolidinium, piperidinium, pyridinium, imidazolium, 1,2,4- and 1,2,3-triazoliums) and anions (e.g., halides, carboxylates, inorganic (per)fluorides or (per)fluorinated sulfonimides) [2,3,16,17]. 1,2,3-triazolium ILs (TILs) have attracted increasing attention over the last decade [18,19,20], as their 1,2,3-triazole precursors constitute essential intermediates that are frequently obtained by the copper(I)-catalyzed azide–alkyne cycloaddition (CuAAC) reaction—the most widespread illustration of click chemistry to date [21,22,23,24]. The structural design of TILs relies on the key attributes of CuAAC (i.e., wide application scope, high yields, functional tolerance and orthogonal reactivity) as well as on the straightforward *N*-alkylation of 1,2,3-triazole intermediates and anion-exchange reactions. TILs have been applied as anticancer drugs [25], antimicrobial agents [26], and in catalysis [27,28], metal extraction [29,30], supramolecular chemistry [31], energy conversion [32], and polymer science [33]. Understanding the correlations between the structure and properties of TILs is crucial to improving their performance and scope of application. In this article, we report on the synthesis of a series of four 1,3,4-trisubstituted TILs with *N*-1-*n*-octyl, *N*-3-methyl and various *C*-4 substituents (i.e., aromatic, aliphatic, PEGylated and fluorinated groups) issued from 1,2,3-triazole precursors in order to contribute to the understanding of the structure–property correlations with the aim of maximizing their ionic conductivity. Firstly, TILs with iodide counter-anions were synthesized in two steps involving: (i) CuAAC reaction between *n*-octyl azide and various alkynes to generate four 1,2,3-triazole precursors; and (ii) the *N*-alkylation of the 1,2,3-triazole groups with methyl iodide. The PEGylated TIL underwent ion metathesis to produce the corresponding TIL with a bis(trifluoromethylsulfonyl)imide counter-anion, which improved its thermal (*T*_d10_), physical (*T*_g_), and ion-conducting (σ_DC_) properties. The correlations between the structures and properties of the synthesized TILs are thoroughly discussed based on characterizations using NMR spectroscopy, high-resolution mass spectrometry, differential scanning calorimetry, thermogravimetric analysis and broadband dielectric spectroscopy.

## 2. Results and Discussion

**Scheme 1:**
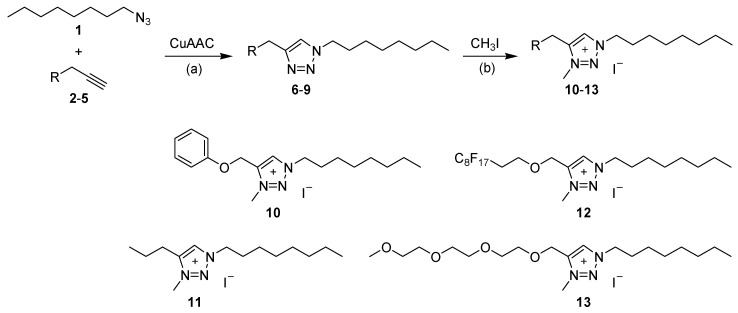
Synthesis of 3-methyl-1,2,3-triazolium iodides **10**-**13**. (**a**): CuI.PO(Et)_3_, DIPEA, THF, 45 °C, 24 h; (**b**): CH_3_CN, 60 °C, 24 h.

**Figure 1:**
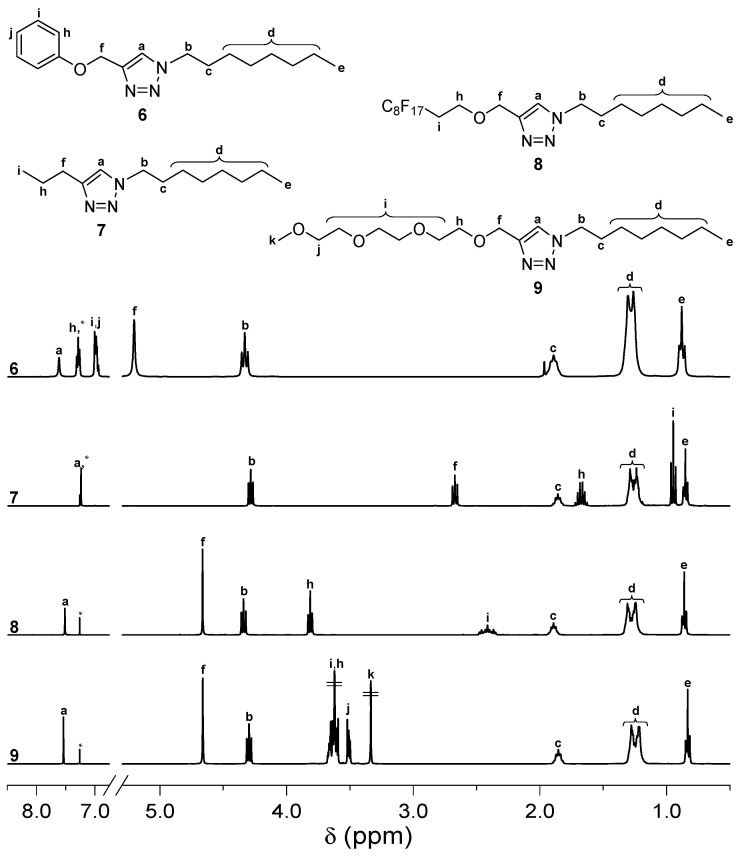
^1^H NMR spectra (400 MHz, 25 °C, CDCl_3_) of 1,2,3-triazoles **6**-**9** (*: Peak assigned to CHCl_3_).

**Figure 2:**
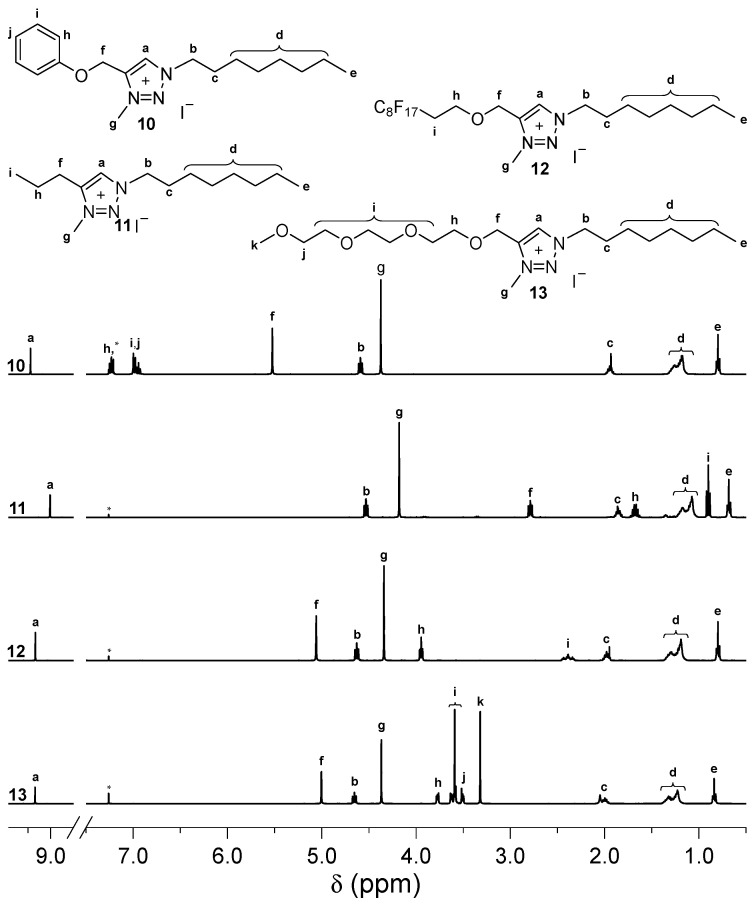
^1^H NMR spectra (400 MHz, 25 °C, CDCl_3_) of 3-methyl-1,2,3-triazolium iodides **10**-**13** (*: Peak assigned to CHCl_3_).

A series of four 1,4-disubstituted 1,2,3-triazoles was first synthesized using copper(I)-catalyzed azide–alkyne cycloaddition (CuAAC) between *n*-octyl azide **1** and terminal alkynes **2**-**5** as the precursors (Scheme 1). Following purification by column chromatography, 1-*n*-octyl-1,2,3-triazoles **6**-**9** were obtained in yields of 88%, 91%, 85% and 82%, respectively, and their structures and purities were confirmed by ^1^H NMR, ^13^C NMR and ^19^F NMR (Figure 1 and Figures S1 and S2). Electrospray-ionization high-resolution mass spectrometry (ESI-HRMS) also confirmed their structures by detecting a single-charge peak corresponding to the [M+H]^+^ adduct for each of them (Table S1). The corresponding 1,3,4-trisubstituted 1-*n*-octyl-3-methyl-1,2,3-triazolium iodides **10**-**13** were then synthesized by *N*-alkylation of the *N*-3 position of 1,2,3-triazoles **6**-**9** using methyl iodide in acetonitrile. They were obtained in almost quantitative yields after the solvent and excess methyl iodide were evaporated under vacuum (Table 1). Combining positive and negative modes ESI-HRMS enabled the detection of single peaks corresponding to, respectively, the 1,2,3-triazolium cations [C]^+^ and [C^+^ + 2I^−^]^−^ adducts, proving the purity of TILs **10**-**13** (Table S1). The ^1^H NMR spectra of 1,2,3-triazolium iodides **10**-**13** (Figure 2) confirmed the quantitative *N*-alkylation of the 1,2,3-triazole groups. This was revealed by the appearance of a single signal corresponding to the 1,2,3-triazolium proton at 9.45, 9.01, 9.34 and 9.34 ppm for **10**-**13**. In contrast, those of 1,2,3-triazoles **6**-**9** were detected at 7.61, 7.23, 7.51 and 7.54 ppm, respectively (Figure 2). Additionally, the appearance of a signal from the *N*-3 methyl protons at 4.37, 4.18, 4.34 and 4.37 ppm for TILs **10**-**13** with an expected 3:1 integration ratio relative to the 1,2,3-triazolium proton was also considered proof of the quantitative *N*-alkylation reaction. Furthermore, the signals of the *N*-1 and *C*-4 methylene groups shifted significantly downfield after the *N*-alkylation reaction due to the higher charge density and electron-withdrawing effect of the 1,2,3-triazolium group compared to the 1,2,3-triazole group. The quantitative N-alkylation was corroborated by comparing the ^13^C NMR spectra of 1,2,3-triazoles **6**-**9** (Figure S1) with those of TILs **10**-**13** (Figure S2). However, while a downfield shift was observed for C-4 methylene groups after N-alkylation, the N-1 methylene groups were shifted upfield. 1,2,3-Triazole **8** and TIL **12** display identical ^19^F NMR spectra (Figure S3).

The phase transition temperatures (*T*_g_ and *T*_m_) of TILs **10**-**13** were investigated using differential scanning calorimetry (DSC, Figure S4) while their thermal stability was assessed by determining their temperature at 10% weight loss (*T*_d10_) using thermogravimetric analysis (TGA, Table 1). The chemical nature of the *C*-4 substituent significantly impacts the physical state of TILs as aromatic TIL **10** and fluorinated TIL **12** are, due to the presence of π-stacking or fluorophilic intermolecular interactions, crystalline solids (*T*_m_ = 69 and 97 °C, respectively), while aliphatic TIL **11** and PEGylated TIL **12** are low *T*_g_ liquids (*T*_g_ = −64 and −60 °C, respectively). These features are consistent with those of previously described TILs with analogous substituents [34,35,36,37]. However, the *C*-4 substituent only moderately affects the thermal stability of TILs, as evidenced by the range of *T*_d10_ values from 185 to 205 °C in the following increasing order: **10** < **12** < **13** < **11** (Figure S5). This is consistent with the known limited thermal stability of halide-containing ILs [38].

**Figure 3:**
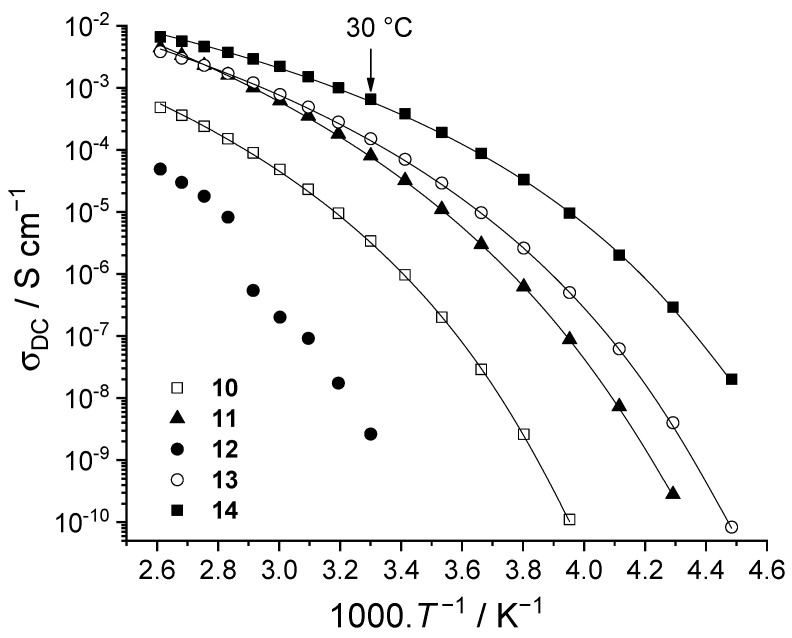
Direct current conductivity (σ_DC_) versus reciprocal temperature for **TILs 10**-**14**. The solid lines represent the best VFT fits of experimental data using Equation (1). σ_∞_, *B* and *T*_0_ parameters to obtain the best fits of experimental data are listed in Table S2.

The temperature dependence of the ionic conductivity of TILs **10**-**13** under anhydrous conditions was studied using broadband dielectric spectroscopy (BDS) and σ_DC_ was plotted as a function of the reciprocal temperature (Figure 3). Except for TIL **12**, the evolution of σ_DC_ with temperature shows a continuous decrease with decreasing temperature. The transition observed for fluorinated TIL **12** is clearly related to its crystallization (*T*_m_ = 97 °C, Table 1), wherein the crystalline phase promotes freezing of the liquid electrolyte and reduces ion transport. However, although TIL **10** is also a crystalline solid (*T*_m_ = 69 °C), no comparable transition was observed in the temperature dependence of σ_DC_. This is most likely due to the slower crystallization kinetics of TIL **10** compared to TIL **12**, which does not allow for crystallization within the timeframe of the BDS measurements. Nevertheless, the σ_DC_ values of TILs **10**-**13**, as measured under anhydrous conditions, depended strongly on the chemical structure of the *C*-4 substituent, and increased in the following order:

σ_DC_ (30 °C, S cm^−1^): **12** (C_8_F_17_CH_2_CH_2_OCH_2_, 2.6 × 10^−9^) << **10** (PhOCH_2_, 3.4 × 10^−6^) < **11** (CH_3_CH_2_CH_2_, 8.0 × 10^−5^) < **13** (CH_3_OCH_2_CH_2_OCH_2_CH_2_OCH_2_CH_2_OCH_2_, 1.5 × 10^−4^)

**Scheme 2:**
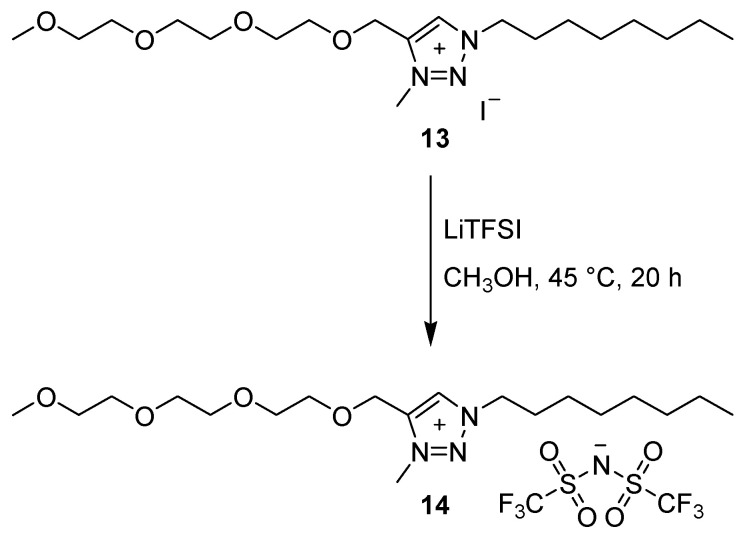
Synthesis of 3-methyl-1,2,3-triazolium bis(trifluoromethylsulfonyl)imide **14**.

Due to its higher ionic conductivity, which is promoted by the flexible triethylene glycol segment [39], TIL **13** underwent an anion-exchange reaction using lithium bis(trifluoromethylsulfonyl)imide, yielding TIL **14** with a bis(trifluoromethylsulfonyl)imide (TFSI) counter-anion (Scheme 2).

The structure and purity of TIL **14** were assessed using ESI-HRMS, ^1^H NMR, ^13^C NMR and ^19^F NMR spectroscopy. The structure was confirmed by the appearance of a single peak corresponding to the 1,2,3-triazolium cation [C]^+^ in positive-mode ESI-HRMS, and the quantitative ion metathesis reaction was corroborated by the detection of a single peak corresponding to the TFSI anion [TFSI]^−^ together to the absence of [I]^−^ and [C^+^ + 2I^−^]^−^ adducts in negative-mode ESI-HRMS (Table S1). Replacing the iodide counter-anion with the larger, asymmetric, more delocalized and weakly coordinating TFSI counter-anion caused the *N*-1/*C*-4 methylene and *N*-3 methyl signals to shift upfield in both the ^1^H and ^13^C NMR spectra (Figures S6 and S7). Furthermore, the presence of the TFSI counter-anion in TIL **14** was confirmed by the appearance of a quadruplet at 119.7 ppm on the ^13^C NMR spectrum of (Figure S7) and a singlet at −78.66 ppm on the ^19^F NMR spectrum (Figure S8). Moreover, the TFSI counter-anion induced a 16 °C decrease in *T*_g_ and a remarkable improvement in thermal stability with a 150 °C increase in *T*_d10_ (Table 1, Figure S5), on a par with previously reported values for TFSI-containing TILs [40,41]. Finally, TIL **14** demonstrated the highest ionic conductivity of the series (σ_DC_ at 30 °C = 6.5 × 10^−4^ S cm^−1^). The evolutions of σ_DC_ with temperature for all TILs but TIL **12** exhibit a Vogel–Fulcher–Tammann (VFT) dependence and were thus fitted using the VFT Equation (1) (Table S2):

σ_DC_ = σ_∞_ × exp(−*B*/(*T* − *T*_0_)) (1)

where σ_∞_ is the ionic conductivity in the limit of high temperatures, *B* is the fitting parameter related to the effective activation energy of ionic conduction, which is related to the energy barrier that the ions must overcome to move from one site to another. For ILs, *B* typically ranges between 800 and 1500 K, corresponding to activation energies between 0.05 and 0.2 eV. *T*_0_, the Vogel temperature, is often interpreted as the ideal glass transition temperature and usually lies 40 K to 60 K below *T*_g_. The higher the value of *T*_0_, the more fragile the ionic conductor is, leading to a higher non-Arrhenius dependence of ionic conductivity as a function of inverse temperature.

## 3. Materials and Methods

### 3.1. Materials

Phenyl propargyl ether (**2**, >90%), 1-pentyne (**3**, 99%), diisopropylethylamine (DIPEA, 99%), copper (I) iodide triethylphosphite (CuI.P(OEt)_3_, 97%), lithium bis(trifluoromethylsulfonyl)imide (LiTFSI, 99.95%), iodomethane (99.5%) and all solvents with the purest grade were purchased from Merck (Darmstadt, Germany) and used as received. *n*-Octyl azide **1** [42], 3,3,4,4,5,5,6,6,7,7,8,8,9,9,10,10,10-heptadecafluorodecyl propargyl ether **4** [43], and 2,5,8,11-tetraoxatetradec-13-yne 5 [44], were synthesized as previously described.

### 3.2. Characterization Methods

^1^H NMR spectra were recorded on a Bruker DRX400 Spectrometer (Billerica, MA, USA) in CDCl_3_ at room temperature. Mass spectra were acquired on a ThermoFinnigan LCQ Advantage ion trap instrument (San Jose, CA, USA), detecting positive (+) or negative (−) ions in the ESI. High-resolution mass spectrometry was performed on all intermediates on a THERMOQUEST Finnigan MAT 95 XL (Waltham, MA, USA) using the “peak matching” method. Differential scanning calorimetry experiments were performed on a DSC Q200 (TA Instruments, New Castle, DE, USA) at a heating rate of 10 °C/min under a helium flow of 25 mL min^−1^. *T*_g_ and *T*_m_ values were measured during the second heating cycle. Thermogravimetric analysis (TGA) was performed on a TGA Q500 apparatus (TA Instruments) at a heating rate of 10 °C min^−1^ and a helium flow of 60 mL min^−1^. Ionic conductivities were measured using a high-resolution Alpha-Analyzer (Novocontrol GmbH, Montabaur, Germany) assisted by a Quatro temperature controller (Richmond, ON, USA). The sample was prepared by placing the ionic liquid between two freshly polished platinum electrodes followed by heating at 120 °C for 4 h under a flow of pure nitrogen. The thickness of the sample cell was controlled by 100 µm thick Teflon spacers (Goodfellow, Lille, France). Frequency sweeps were performed isothermally from 10 MHz to 0.1 Hz by applying a sinusoidal voltage of 0.1 V over a range of temperature from 120 to −60 °C. The temperature was controlled by heating the sample under flow of pure nitrogen, which excludes the presence of oxygen and humidity in the measurement chamber. The thermal stability was set to be better than 0.1 K in absolute values with relative variations less than 0.2 K min^−1^.

### 3.3. General Procedure for the Synthesis of 1-n-Octyl-1,2,3-Triazoles by Copper(I)-Catalyzed Azide–Alkyne 1,3-Dipolar Cycloaddition

Synthesis of **6**. CuI.P(OEt)_3_ (0.06 g, 0.2 mmol) and DIPEA (1.97 g, 15.2 mmol) were added to a solution of *n*-octyl azide **1** (2.35 g, 15.2 mmol) and phenyl propargyl ether **2** (2.00 g, 15.2 mmol) in tetrahydrofuran (50 mL). The mixture was stirred under argon for 24 h at 45 °C before being evaporated to dryness under reduced pressure. The crude product was purified by column chromatography using a 3:2 mixture of petroleum ether and ethyl acetate to yield after evaporation of the solvents under reduced pressure **6** as an off-white crystalline solid (3.83 g, Yield 88%). ^1^H NMR (400 MHz, CDCl_3_, δ, ppm): 7.61 (s, 1H), 7.31–7.25 (m, 2H), 7.08–6.93 (m, 3H), 5.20 (s, 2H), 4.33 (t, *J* = 7.5 Hz, 2H), 1.96–1.87 (m, 2H), 1.40–1.26 (m, 10H), 0.88 (t, *J* = 6.9 Hz, 3H). ^13^C NMR (100 MHz, CDCl_3_, δ, ppm): 158.0, 129.3, 121.0, 120.6, 114.6, 61.8, 50.3, 31.5, 30.1, 29.0-28.7, 26.3, 22.4, 13.9. ESI-HRMS (*m*/*z*): [M + H]^+^ calcd for C_17_H_26_N_3_O, 288.2070; Found, 288.2067.

Synthesis of **7**. The general procedure for copper(I)-catalyzed azide–alkyne 1,3-dipolar cycloaddition was applied to a mixture of *n*-octyl azide **1** (2.50 g, 16.1 mmol), 1-pentyne **3** (1.10 g, 16.1 mmol), CuI.P(OEt)_3_ (0.08 g, 0.21 mmol) and DIPEA (2.08 g, 16.1 mmol) in tetrahydrofuran (50 mL). The crude pale brown oily product was purified by column chromatography using a 4:1 mixture of petroleum ether and ethyl acetate to yield after evaporation of the solvents under reduced pressure **7** as a brown liquid (3.27 g, Yield 91%). ^1^H NMR (400 MHz, CDCl_3_, δ, ppm): 7.23 (s, 1H), 4.28 (t, *J* = 7.3 Hz, 2H), 2.67 (t, *J* = 7.6 Hz, 2H), 1.89–1.82 (m, 2H), 1.72–1.62 (m, 2H), 1.34-1.17 (m, 10H), 0.95 (t, *J* = 7.6 Hz, 3H), 0.88 (t, *J* = 6.9 Hz, 3H). ^13^C NMR (100 MHz, CDCl_3_, δ, ppm): 148.1, 120.4, 50.1, 31.7, 30.3, 29.0–28.9, 27.7, 22.7, 22.6, 14.0, 13.7. ESI-HRMS (*m*/*z*): [M + H]^+^ calcd for C_13_H_26_N_3_, 224.2121; Found, 224.2115.

Synthesis of **8**. The general procedure for copper(I)-catalyzed azide–alkyne 1,3-dipolar cycloaddition was applied to a mixture of *n*-octyl azide **1** (2.50 g, 16.1 mmol), 7,7,8,8,9,9,10,10,11,11,12,12,13,13,14,14,14-heptadecafluoro-4-oxatetradeca-1-yne **4** (8.08 g, 16.1 mmol), CuI.P(OEt)_3_ (0.08 g, 0.21 mmol) and DIPEA (2.08 g, 16.1 mmol) in tetrahydrofuran (50 mL). The crude pale brown oily product was purified by column chromatography using a 1:1 mixture of methylene chloride and ethyl acetate to yield after evaporation of the solvents under reduced pressure **8** as a brown liquid (9.01 g, Yield 85%). ^1^H NMR (400 MHz, CDCl_3_, δ, ppm): 7.51 (s, 1H), 4.66 (s, 2H), 4.34 (t, *J* = 7.6 Hz, 2H), 3.81 (t, *J* = 7.5 Hz, 2H), 2.48-2.32 (m, 2H), 1.89 (m, 2H), 1.34–1.20 (m, 10H), 0.86 (t, *J* = 6.9 Hz, 3H). ^13^C NMR (100 MHz, CDCl_3_, δ, ppm): 144.5, 122.2, 122.0-105.0, 64.6, 62.2, 50.4, 31.7, 31.4, 30.3, 29.0–28.9, 26.4, 22.5, 14.0. ^19^F NMR (376 MHz, CDCl_3_, δ, ppm): −80.92 (s, 3F), −113.49 (s, CF3CF2CF2CF2CF2CF2CF2CF2CF2, 2F), −121.79 (s, 2F), −122.04 (s, 4F), −122.84 (s, 2F), −123.74 (s, 2F), −126.25 (s, 2F). ESI-HRMS (*m*/*z*): [M + H]^+^ calcd for C_21_H_25_F_17_N_3_O, 658.1721; Found, 658.1714.

Synthesis of **9**. The general procedure for copper(I)-catalyzed azide–alkyne 1,3-dipolar cycloaddition was applied to a mixture of *n*-octyl azide **1** (2.50 g, 16.1 mmol), 2,5,8,11-tetraoxatetradec-13-yne **5** (3.25 g, 16.1 mmol), CuI.P(OEt)_3_ (0.08 g, 0.21 mmol) and DIPEA (2.08 g, 16.1 mmol) in tetrahydrofuran (50 mL). The crude pale brown oily product was purified by column chromatography using a 4:1 mixture of petroleum ether and ethyl acetate to yield after evaporation of the solvents under reduced pressure **9** as brown liquid (4.72 g, Yield 82%). ^1^H NMR (400 MHz, CDCl_3_, δ, ppm): 7.54 (s, 1H), 4.66 (s, 2H), 4.30 (t, *J* = 7.3 Hz, 2H), 3.69-3.58 (m, 10H), 3.52–3.50 (m, 2H), 3.34 (s, 3H), 1.86 (m, 2H), 1.31–1.18 (m, 10H), 0.83 (t, *J* = 6.9 Hz, 3H). ^13^C NMR (100 MHz, CDCl_3_, δ, ppm): 145.0, 122.3, 71.8, 70.6-70.4, 69.6, 64.6, 58.9, 50.3, 31.6, 30.2, 28.9-28.8, 26.4, 22.5, 14.0. ESI-HRMS (*m*/*z*): [M + H]^+^ calcd for C_18_H_36_N_3_O_4_, 358.2700; Found, 358.2709.

### 3.4. General Procedure for the Synthesis of 1-n-Octyl-3-Methyl-1,2,3-Triazolium Iodides by N-Alkylation of 1,2,3-Triazoles

Synthesis of **10**. Iodomethane (8.13 mL, 130.5 mmol) was added drop wise to a solution of **6** (3.75 g, 13.1 mmol) in acetonitrile (50 mL) and the reaction mixture was stirred for 24 h at 60 °C. Solvent and excess iodomethane were evaporated under reduced pressure to yield **10** as an off-white crystalline solid (5.32 g, Yield 95%). ^1^H NMR (400 MHz, CDCl_3_, δ, ppm): 9.45 (s, 1H), 7.28–7.18 (m, 2H), 7.01–6.91 (m, 3H), 5.52 (s, 2H), 4.59 (t, *J* = 7.5 Hz, 2H), 4.37 (s, 3H), 1.99–1.90 (m, 2H), 1.34–1.13 (m, 10H), 0.80 (t, *J* = 6.9 Hz, 3H). ^13^C NMR (100 MHz, CDCl_3_, δ, ppm): 156.4, 139.4, 130.7, 129.6, 122.3, 114.7, 58.8, 54.2, 39.9, 31.4, 29.2, 28.8–28.5, 25.8, 22.3, 13.8. ESI-HRMS (*m*/*z*): [C]^+^ calcd for C_18_H_28_N_3_O, 302.2227; Found, 302.2222. [C^+^ + 2I^−^]^−^ calcd for C_18_H_28_I_2_N_3_O, 556.0327; Found, 556.0337.

Synthesis of **11**. The general procedure for *N*-alkylation of 1,2,3-triazoles was applied to a mixture of **7** (3.00 g, 13.4 mmol) and iodomethane (8.37 mL, 134.3 mmol) in acetonitrile (50 mL) yielding after evaporation of the solvent and excess iodomethane **11** as a brown viscous oil (4.70 g, Yield 96%). ^1^H NMR (400 MHz, CDCl_3_, δ, ppm): 9.01 (s, 1H), 4.53 (t, *J* = 7.5 Hz, 2H), 4.18 (s, 3H), 2.79 (t, *J* = 7.6 Hz, 2H), 1.93–1.82 (m, 2H), 1.74–1.62 (m, 2H), 1.26–1.00 (m, 10H), 0.90 (t, *J* = 7.6 Hz, 3H), 0.68 (t, *J* = 6.9 Hz, 3H). ^13^C NMR (100 MHz, CDCl_3_, δ, ppm): 143.8, 128.7, 53.6, 38.6, 31.0, 28.9, 28.3-28.1, 25.5, 25.1, 21.9, 20.1, 13.5, 13.1. ESI-HRMS (*m*/*z*): [C]^+^ calcd for C_14_H_28_I_2_N_3_, 238.2278; Found, 238.2273. [C^+^ + 2I^−^]^−^ calcd for C_14_H_28_I_2_N_3_, 492.0377; Found, 492.0367.

Synthesis of **12**. The general procedure for *N*-alkylation of 1,2,3-triazoles was applied to a mixture of **8** (8.60 g, 13.1 mmol) and iodomethane (8.14 mL, 130.6 mmol) in acetonitrile (50 mL), yielding after evaporation of the solvent and excess iodomethane **12** as a brown viscous oil (9.95 g, Yield 95%). ^1^H NMR (400 MHz, CDCl_3_, δ, ppm): 9.34 (s, 1H), 5.06 (s, 2H), 4.63 (t, *J* = 7.6 Hz, 2H), 4.34 (s, 3H), 3.95 (t, *J* = 7.5 Hz, 2H), 2.45-2.36 (m, 2H), 1.95 (m, 2H), 1.35–1.17 (m, 10H), 0.80 (t, *J* = 6.9 Hz, 3H). ^13^C NMR (100 MHz, CDCl_3_, δ, ppm): 139.8, 130.5, 122.7–104.8, 63.5, 60.9, 54.3, 39.5, 31.4, 31.0, 29.3, 28.7–28.6, 25.9, 22.3, 13.8. ^19^F NMR (376 MHz, CDCl_3_, δ, ppm): −81.05 (s, 3F), −113.49 (s, 2F), −121.84 (s, 2F), −122.14 (s, 4F), −122.95 (s, 2F), −123.74 (s, 2F), −126.37 (s, 2F). ESI-HRMS (*m*/*z*): [C]^+^ calcd for C_22_H_27_F_17_N_3_O, 672.1877; Found, 672.1862. [C^+^ + 2I^−^]^−^ calcd for C_22_H_27_F_17_I_2_N_3_O, 925.9978; Found, 925.9953.

Synthesis of **13**. The general procedure for *N*-alkylation of 1,2,3-triazoles was applied to a mixture of **9** (4.45 g, 12.4 mmol) and iodomethane (7.74 mL, 124.3 mmol) in acetonitrile (45 mL), yielding after evaporation of the solvent and excess iodomethane **13** as a brown viscous oil (5.90 g, Yield 95%). ^1^H NMR (400 MHz, CDCl_3_, δ, ppm): 9.34 (s, 1H), 5.00 (s, 2H), 4.65 (t, *J* = 7.3 Hz, 2H), 4.37 (s, 3H), 3.78-3.76 (m, 2H), 3.63-3.56 (m, 8H), 3.52-3.49 (m, 2H), 3.32 (s, 3H), 2.00 (m, 2H), 1.34–1.20 (m, 10H), 0.84 (t, *J* = 6.9 Hz, 3H). ^13^C NMR (100 MHz, CDCl_3_, δ, ppm): 140.4, 130.6, 71.7, 70.5–69.9, 60.8, 58.8, 54.2, 39.3, 31.5, 29.4, 28.8–28.7, 26.0, 22.5, 14.0. ESI-HRMS (*m*/*z*): [C]^+^ calcd for C_19_H_38_N_3_O_4_, 372.2857; Found, 372.2858. [C^+^ + 2I^−^]^−^ calcd for C_19_H_38_I_2_N_3_O_4_, 626.0957; Found, 626.0975.

### 3.5. Synthesis of 1-n-Octyl-3-Methyl-1,2,3-Triazolium 14 by Ion-Exchange Reaction

A solution of **13** (1.26 g, 2.59 mmol) and lithium bis(trifluoromethylsulfonyl)imide (0.78 g, 2.73 mmol) in methanol (15 mL) was heated for 20 h at 45 °C. The solution was evaporated to dryness under vacuum, the crude product was dissolved in water (10 mL) and extracted three times with dichloromethane (3 × 50 mL). The organic phase was dried with MgSO_4_, filtered and passed through a thin layer of neutral alumina. The resulting solution was evaporated to dryness under reduced pressure to give **14** as a brown liquid (1.25 g, Yield 74%). ^1^H NMR (400 MHz, CDCl_3_, δ, ppm): 8.45 (s, 1H), 4.77 (s, 2H), 4.47 (t, *J* = 7.4 Hz, 2H), 4.25 (s, 3H), 3.68–3.64 (m, 2H), 3.63–3.52 (m, 8H), 3.51–3.47 (m, 2H), 3.29 (s, 3H), 1.95–1.92 (m, 2H), 1.38–1.22 (m, 10H), 0.84 (t, *J* = 6.6 Hz, 3H). ^13^C NMR (100 MHz, CDCl_3_, δ, ppm): 140.6, 129.3, 119.7 (q, *J* = 321.5 Hz, 2C), 71.7, 70.4–70.0, 60.1, 58.6, 54.0, 39.4, 31.4, 29.0, 28.7–28.5, 26.0, 25.9, 22.3, 13.8. ^19^F NMR (376 MHz, CDCl_3_, δ, ppm): −78.66 (s, 6F). ESI-HRMS (*m*/*z*): [C]^+^ calcd for C_19_H_38_N_3_O_4_, 372.2857; Found, 372.2854. [TFSI]^−^ calcd for C_2_F_6_NO_4_S_2_, 279.9178; Found, 279.9179.

## 4. Conclusions

We have reported a straightforward route for synthesizing of 1,3,4-trisubstituted-1,2,3-triazolium ionic liquids by combining the robust features of CuAAC click chemistry with *N*-alkylation of 1,2,3-triazole groups and ion-exchange reactions. The physical state of the resulting TILs (*T*_g_ and *T*_m_) was strongly impacted by variation in the C-4 substituent. Their thermal stability and ion-conducting properties were particularly affected by the nature of the counter-anion. The most desirable properties (*T*_g_ = −76 °C, *T*_d10_ = 345 °C, σ_DC_ at 30 °C = 6.5 × 10^−4^ S cm^−1^) were achieved with the TFSI counter-anion and the PEGylated C-4 substituent. This versatile strategy demonstrates high functional tolerance and orthogonality. It is therefore anticipated that it will contribute to the further expansion of the range of task-specific TILs using functional synthetic precursors. The wide structural design related to the chemical structures of the counter anion and the *N*-1 and *C*-4 substituents allows applications involving ionic conductivity, phase separation, lubrication, rheology, catalysis and surface properties to be addressed.

## Figures and Tables

**Table 1:** Physical properties of 1,2,3-triazolium ionic liquids **10**-**14**.

TIL	Anion	Yield (%)	*T*_g_ (°C) ^[a]^	*T*_m_ (°C) ^[a]^	*T*_d10_ (°C) ^[b]^	σ_DC_ (S cm^−1^) ^[c]^
**10**	I	95	−26	69	185	3.4 × 10^−6^
**11**	I	96	−64	/	205	8.0 × 10^−5^
**12**	I	95	3	97	190	2.6 × 10^−9^
**13**	I	95	−60	/	195	1.5 × 10^−4^
**14**	TFSI	74	−76	/	345	6.5 × 10^−4^

^[a]^ Measured by DSC. ^[b]^ Measured by TGA. ^[c]^ Measured at 30 °C under anhydrous conditions by BDS.

## Data Availability

The original contributions presented in this study are included in the article and Supporting Information. Further inquiries can be directed to the corresponding authors.

## Supplementary Materials

The following supporting information can be downloaded at: https://www.mdpi.com/article/10.3390/molecules31060936/s1, Figures S1–S3, S6–S8: ^1^H, ^13^C and ^19^F NMR spectra of 1,2,3-triazoles **6**-**9** and 1,2,3-triazoliums **10**-**14**; Figure S4: DSC traces of 1,2,3-triazoliums **10**-**14**; Figure S5: TGA traces of 1,2,3-triazoliums **10**-**14**; Table S1: ESI-HRMS data for of 1,2,3-triazoles **6**-**9** and 1,2,3-triazoliums **10**-**14**; Table S2: VFT fitting parameters of the temperature dependence of the ionic conductivity of 1,2,3-triazoliums **10**,**11**,**13**,**14**.

## Abbreviations

The following abbreviations are used in this manuscript:

BDS	Broadband dielectric spectroscopy
CuAAC	Copper(I)-catalyzed azide–alkyne 1,3-dipolar cycloaddition
DSC	Differential scanning calorimetry
EMIM	1-Ethyl-3-methylimidazolium
IL	Ionic liquid
NMR	Nuclear magnetic resonance
PEG	Poly(ethylene glycol)
TFSI	bis(trifluoromethylsulfonyl)imide
TGA	Thermogravimetric analysis
TIL	1,2,3-Triazolium ionic liquids
