# Supplementary material for: Synthesis, Physical and Ion-Conducting Properties of 1,2,3-Triazolium Ionic Liquids
Source: Molecules. 2026 Mar 11;31(6):936. doi: 10.3390/molecules31060936 (PMC13029745; doi:10.3390/molecules31060936)
Supplement: Supplementary file 1 [file molecules-31-00936-s001.zip › molecules-4185019-supplementary.pdf]

Supplementary Information

Synthesis, Physical and Ion Conducting Properties of 1,2,3-Triazolium Ionic Liquids

Imen Abdelhedi Miladi [1,*], Maha Chikhaoui [1,2], Malak Alaa Eddine [2], Anatoli Serghei [2], Hatem Ben Romdhane [1] and Eric Drockenmuller [2,*]

[1] Université de Tunis El Manar, Faculté des Sciences de Tunis, Laboratoire de Chimie (Bio)Organique Structurale et de Polymères (LR99ES14), 2092 El Manar, Tunisia

[2] Université Claude Bernard Lyon 1, CNRS, Ingénierie des Matériaux Polymères, UMR 5223, Villeurbanne 69622, France

* Correspondence: imen.abdelhedi@fst.utm.tn (I.A.M.); eric.drockenmuller@univ-lyon1.fr (E.D.)

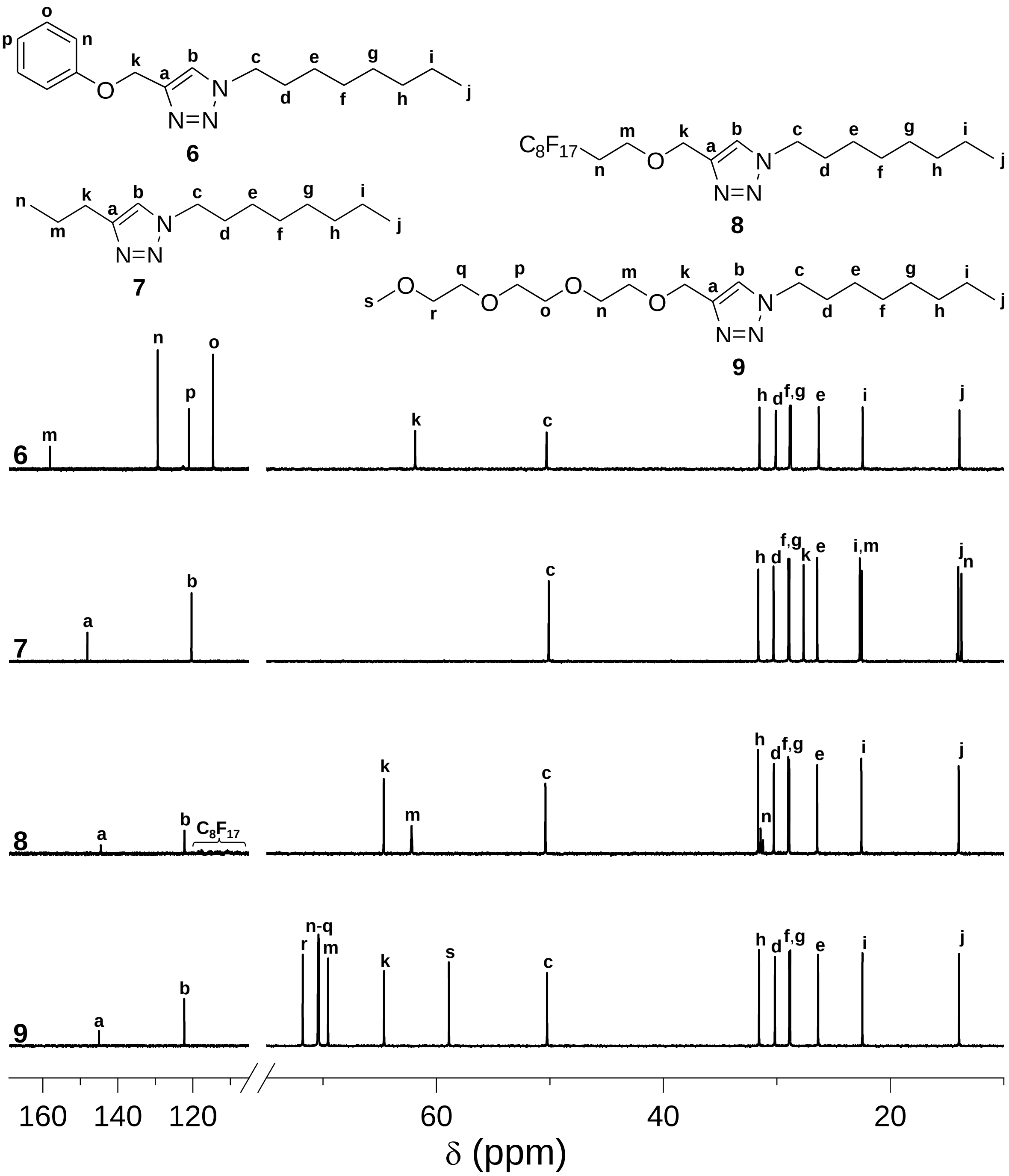

Figure S1. ^{13}C NMR spectra (400 MHz, 25 °C, $CDCl_3$) of 1,2,3-triazoles **6**-**9**.

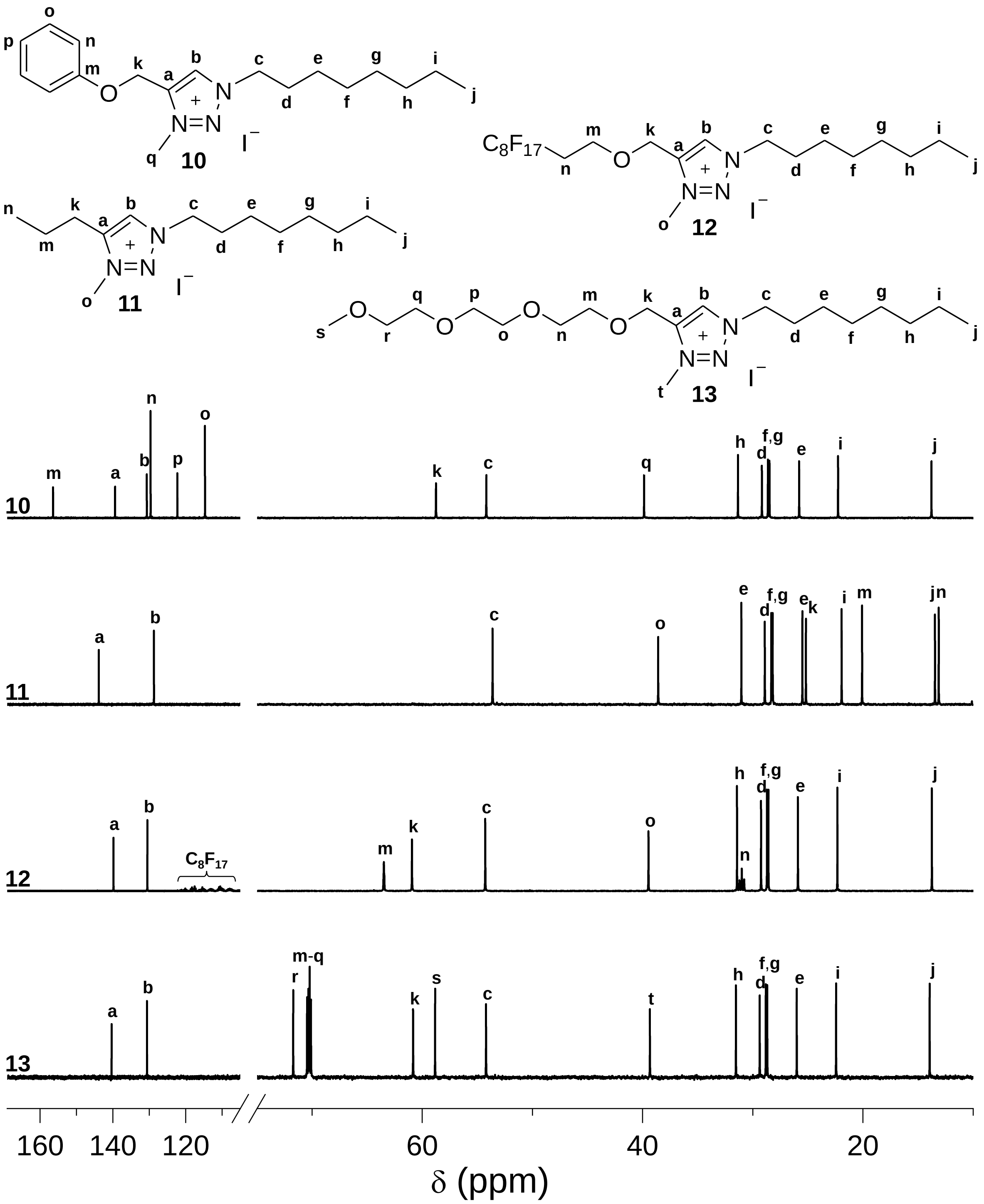

Figure S2. ^{13}C NMR spectra (100 MHz, 25 °C, $CDCl_3$) of 3-methyl-1,2,3-triazoliums **10**-**13**.

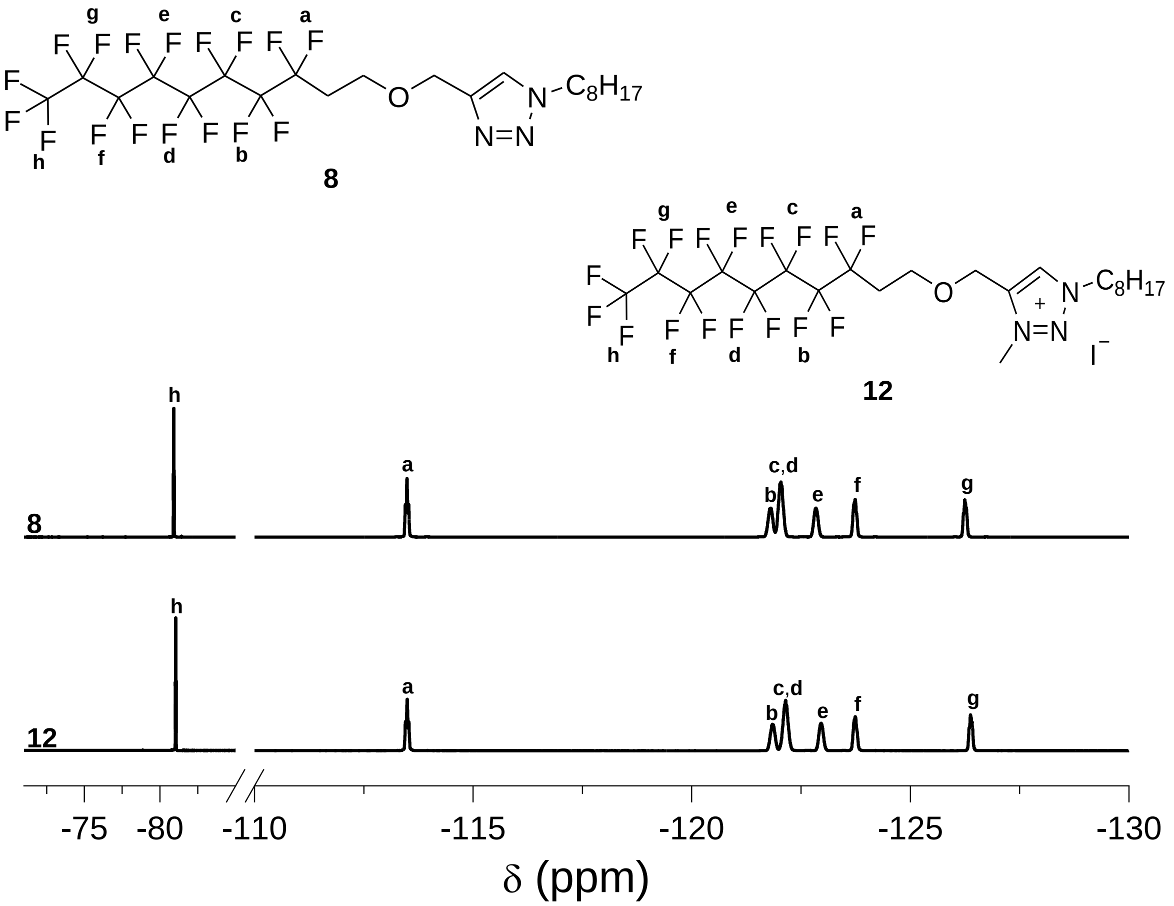

Figure S3. ^{19}F NMR spectra (376 MHz, 25 °C, $CDCl_3$) of 1,2,3-triazole **8** and 3-methyl-1,2,3-triazolium **12**.

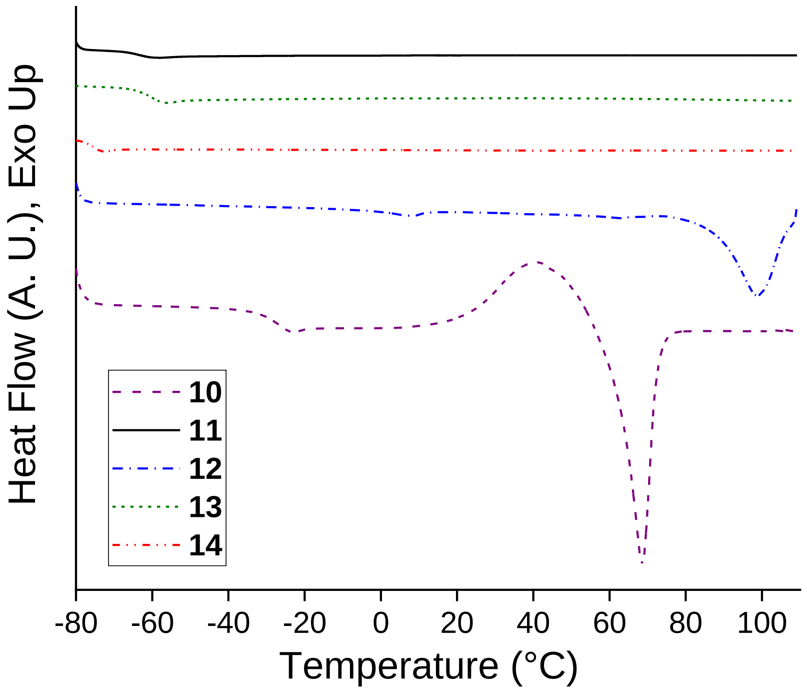

Figure S4. DSC traces of 3-methyl-1,2,3-triazoliums **10**-**14**.

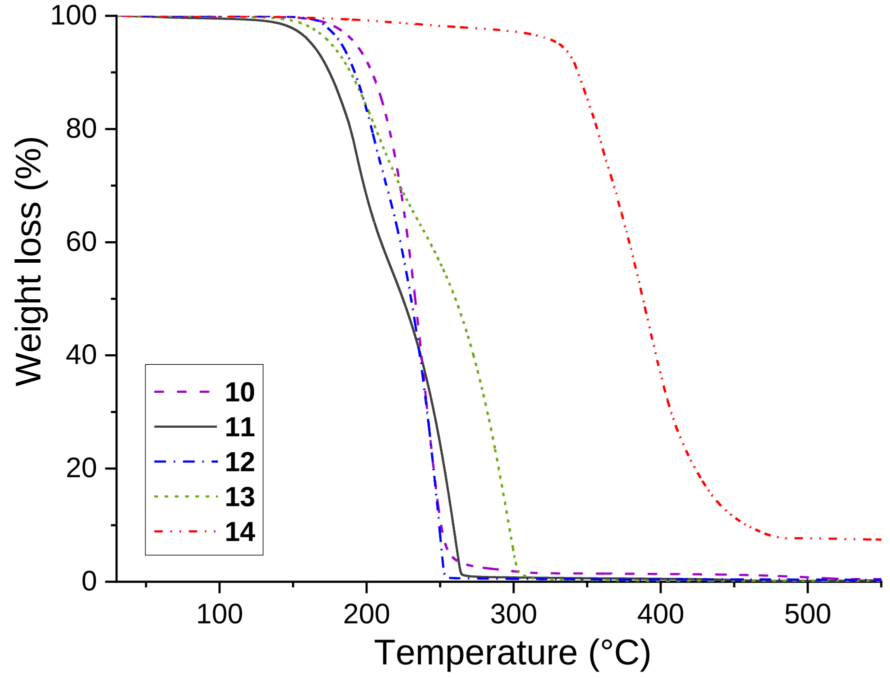

Figure S5. TGA traces of 3-methyl-1,2,3-triazoliums **10-14**.

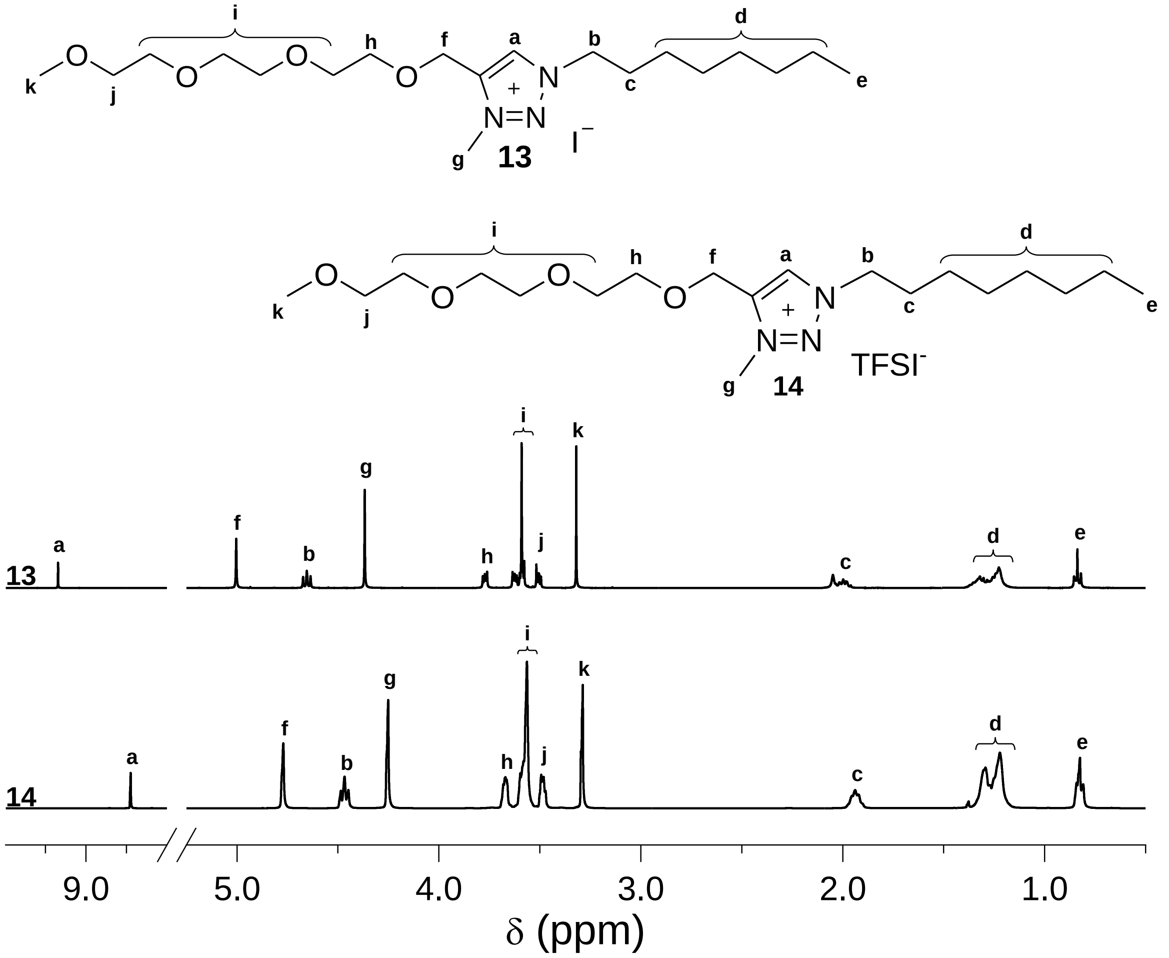

Figure S6. ^{1}H NMR spectra (400 MHz, 25 °C, $CDCl_3$) of 3-methyl-1,2,3-triazoliums **13** and **14**.

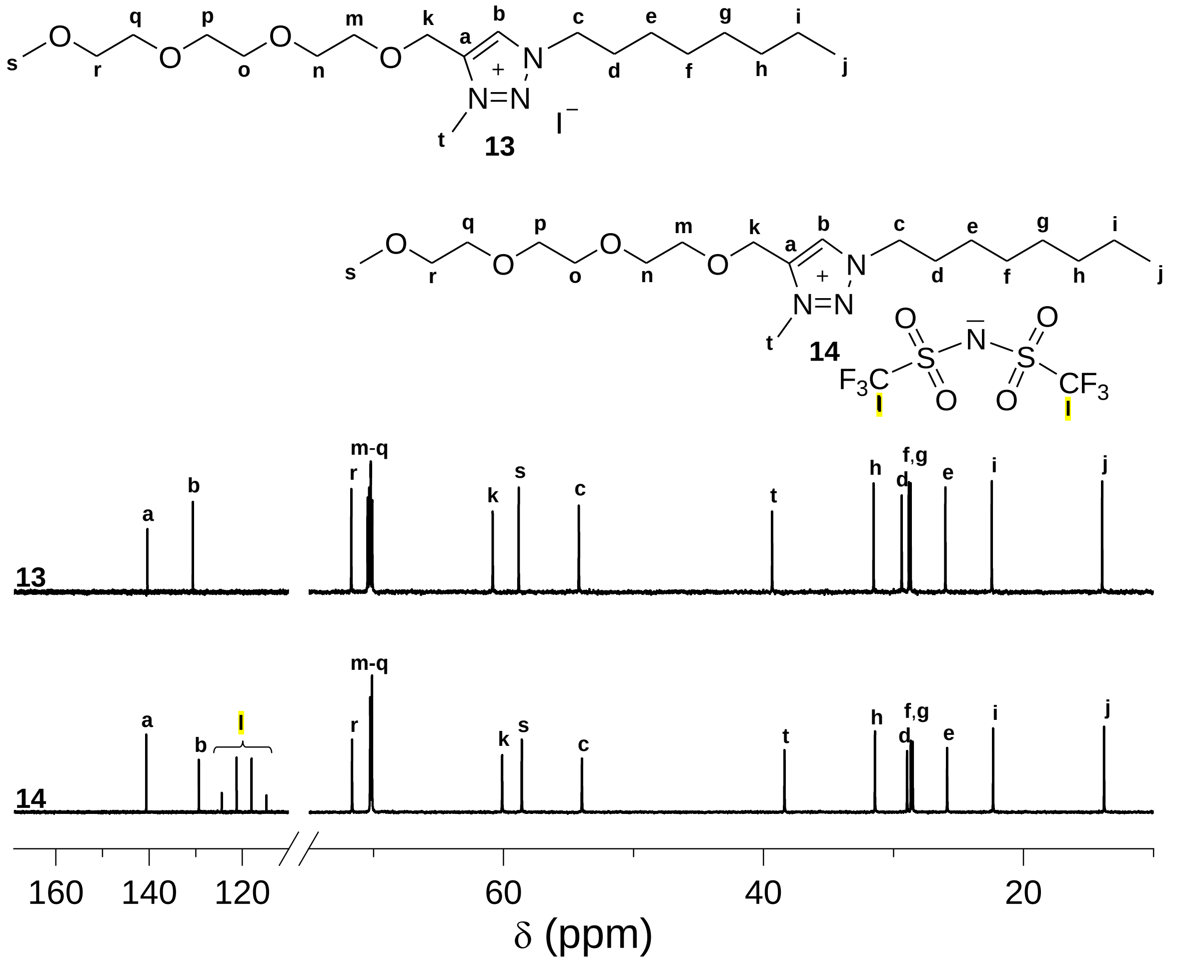

Figure S7. ^{13}C NMR spectra (100 MHz, 25 °C, $CDCl_3$) of 3-methyl-1,2,3-triazoliums **13** and **14**.

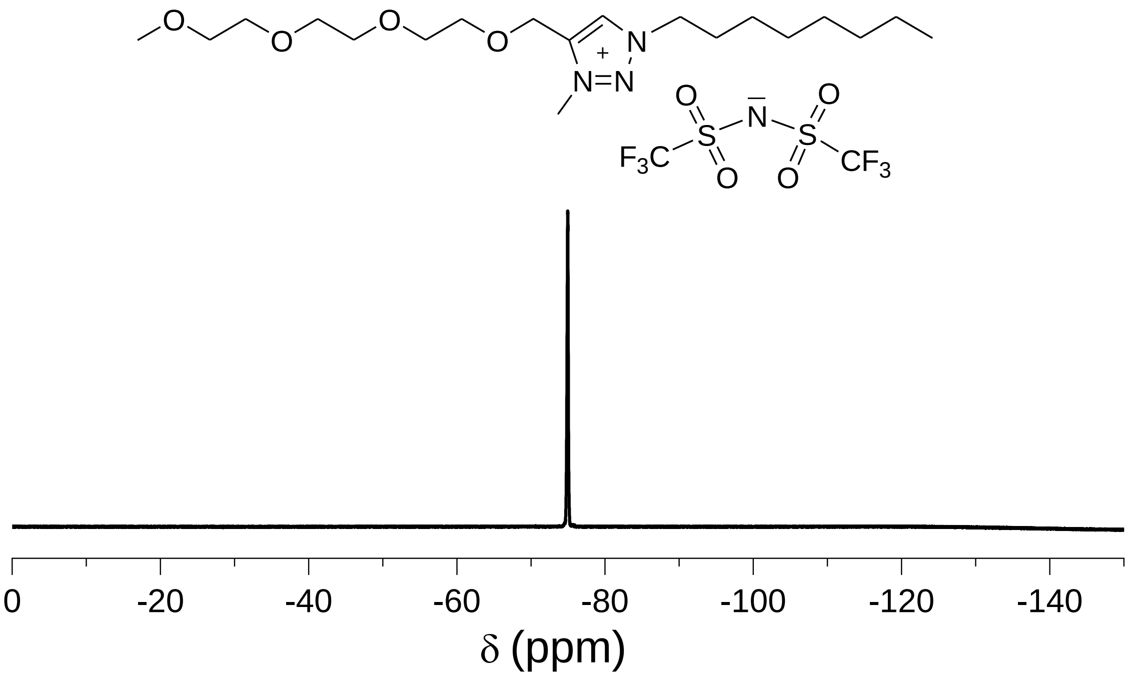

Figure S8. ^{19}F NMR spectrum (376 MHz, 25 °C, $CDCl_3$) of 3-methyl-1,2,3-triazolium **14**.

Table S1. Positive and negative modes ESI-HRMS characterization of 1,2,3-triazoles **6-9** and 1,2,3-triazoliums **10-14**.

Entry	Major peak	Charge (z)	Raw formula of ionic fragment	Theoretical mass (m/z)	Experimental mass (m/z)
**6**	$[M+H]^+$	+1	$C_{17}H_{26}N_3O$	288.2070	288.2067
**7**	$[M+H]^+$	+1	$C_{13}H_{26}N_3$	224.2121	224.2115
**8**	$[M+H]^+$	+1	$C_{21}H_{25}F_{17}N_3O$	658.1721	658.1714
**9**	$[M+H]^+$	+1	$C_{18}H_{36}N_3O_4$	358.2700	358.2709
**10**	$[C]^+$	+1	$C_{18}H_{28}N_3O$	302.2227	302.2222
	$[C^+ + 2I^-]^-$	–1	$C_{18}H_{28}I_2N_3O$	556.0327	556.0337
**11**	$[C]^+$	+1	$C_{14}H_{28}N_3$	238.2278	238.2273
	$[C^+ + 2I^-]^-$	–1	$C_{14}H_{28}I_2N_3$	492.0378	492.0377
**12**	$[C]^+$	+1	$C_{22}H_{27}F_{17}N_3O$	672.1877	672.1862
	$[C^+ + 2I^-]^-$	–1	$C_{22}H_{27}F_{17}I_2N_3O$	925.9978	925.9953
**13**	$[C]^+$	+1	$C_{19}H_{38}N_3O_4$	372.2857	372.2858
	$[C^+ + 2I^-]^-$	–1	$C_{19}H_{38}I_2N_3O_4$	626.0957	626.0975
**14**	$[C]^+$	+1	$C_{19}H_{38}N_3O_4$	372.2857	372.2854
	$[TFSI]^-$	–1	$C_2F_6NO_4S_2$	279.9178	279.9189

Table S2. VFT fitting parameters and ion conducting properties of 1,2,3-triazoliums **10-14**.

Entry	σ_∞ (S cm^{-1})[a]	B (K)[a]	T_0 (K)[a]	$T_g - T_0$ (K)[a]	σ_{DC} at 30 °C (S cm^{-1})[b]	σ_{DC} at 90 °C (S cm^{-1})[b]
**10**	0.55	1301	195	52	3.4×10^{-6}	2.4×10^{-4}
**11**	3.85	1406	173	36	8.0×10^{-5}	2.4×10^{-3}
**12**	/	/	/	/	2.6×10^{-9}	1.8×10^{-5}
**13**	1.30	1146	174	39	1.5×10^{-4}	2.4×10^{-3}
**14**	0.44	861	172	25	6.5×10^{-4}	4.8×10^{-3}

[a] Obtained from VFT fits of the experimental data. [b] Measured under anhydrous conditions by BDS.
